# Endo-IP and lyso-IP toolkit for endolysosomal profiling of human-induced neurons

**DOI:** 10.1073/pnas.2419079121

**Published:** 2024-12-05

**Authors:** Frances V. Hundley, Miguel A. Gonzalez-Lozano, Lena M. Gottschalk, Aslan N. K. Cook, Jiuchun Zhang, Joao A. Paulo, J. Wade Harper

**Affiliations:** ^a^Department of Cell Biology, Harvard Medical School, Boston, MA 02115; ^b^Aligning Science Across Parkinson’s Collaborative Research Network, Chevy Chase, MD 20815; ^c^Initiative in Trafficking and Neurodegeneration, Department of Cell Biology, Harvard Medical School, Boston, MA 02115

**Keywords:** endosome, proteomics, lysosome, iNeuron, stem cells

## Abstract

Plasma membrane protein degradation and recycling are regulated by the endolysosomal system, wherein endocytic vesicles bud from the plasma membrane into the cytoplasm and mature into endosomes and then degradative lysosomes. As such, the endolysosomal system plays a critical role in determining the abundance of proteins on the cell surface and influencing cellular identity and function. Highly polarized cells, like neurons, rely on the endolysosomal system for axonal and dendritic specialization and synaptic compartmentalization. The importance of this system to neuronal function is reflected by the prevalence of risk variants in components of the system in several neurodegenerative diseases, ranging from Parkinson’s to Alzheimer’s disease. Nevertheless, our understanding of endocytic cargo and core endolysosomal machinery in neurons is limited, in part due to technical limitations. Here, we develop a toolkit for capturing EEA1-positive endosomes (termed Endo-IP) and TMEM192-positive lysosomes (termed Lyso-IP) in stem cell-derived induced neurons (iNeurons). We demonstrate its utility by revealing the endolysosomal protein landscapes for stem cells and cortical-like iNeurons, and profiling endosomes in response to potassium-mediated neuronal depolarization. Through global profiling of endocytic cargo, we identify hundreds of transmembrane proteins, including neurogenesis and synaptic proteins, as well as endocytic cargo with predicted SNX17 or SNX27 recognition motifs. By contrast, parallel lysosome profiling reveals a simpler protein repertoire, reflecting in part temporally controlled recycling or degradation for many endocytic targets. This system will facilitate mechanistic interrogation of endolysosomal components found as risk factors in neurodegenerative disease.

## Significance

Approximately one-third of human genes encode proteins containing transmembrane segments. The abundance of many of these proteins, particularly those localized on the plasma membrane (PM), is controlled by the endolysosomal system through the process of endocytosis. During this process, proteins are captured by endosomes and sorted for recycling to the PM or Golgi, or alternatively, targeted for lysosomal degradation. Defects in these pathways are linked to neurodegenerative diseases like Parkinson's. However, the complete repertoire of endocytic cargo and regulatory pathways, particularly in neurons, remains poorly understood. Here, we develop a genetic toolkit that facilitates analysis of early endosomes and lysosomes in stem cell-derived neurons and demonstrate its utility in defining the landscape of endocytic cargo in cortical-like iNeurons.

The endolysosomal system plays a critical role in controlling proteome abundance and quality in eukaryotic cells. First, specific plasma membrane (PM) proteins—referred to here as cargo—undergo the process of endocytosis, where they are concentrated into an inwardly budding membrane structure to form an endocytic vesicle, which matures to an endosome. Endosomes coordinate three primary protein trafficking systems (1–6): 1) recycling of cargo proteins back to the PM via sorting into recycling endosomes, 2) retrograde transport of cargo proteins to the Golgi, allowing recycling through the secretory pathway, and 3) trafficking into late endosomal intralumenal vesicles via the ESCRT pathway, ultimately leading to cargo degradation upon maturation into fully degradative lysosomes. These mechanisms collaborate to maintain and dynamically modulate the cell surface proteome while also coordinating intracellular signaling of multiple classes of ligand-activated receptors. Second, the endolysosomal system plays a key role in proteome quality control through the process of autophagy (7). Here, intracellular organelles and proteins are marked for capture within an autophagosome, which then fuse with degradative lysosomes, resulting in degradation of the cargo.

Arguably, among the most demanding settings for spatial control of the endolysosomal system are highly polarized cells such as neurons, which consist of complex dendritic arbors and axons that extend many times the length of the cell body (soma) (8–11). Regulated abundance of PM proteins controls numerous aspects of neuronal function, ranging from axon guidance to the identity and function of pre- and postsynaptic compartments. The distinct dendritic and axonal cohorts of PM proteins are controlled by the axon initial segment and regulated endocytic degradation (12–14). Moreover, organelle quality control via autophagy especially within axons relies on the formation of endolysosomes which mature to form lysosomes that are capable of fusion with autophagosomes distally (15). Such autolysosomes are subsequently trafficked back to the soma and become sufficiently acidic for support of cargo degradation during transit to the soma (16). The importance of endolysosomal trafficking for neuronal health is indicated by the number and diversity of genes in the pathway that are risk variants across neurodegenerative diseases, ranging from Parkinson’s to Alzheimer’s diseases, including LRRK2, VPS35, BIN1, and PSEN1/2 (17–21). Defects in such pathways may alter the location and abundance of critical PM proteins, as demonstrated for the VPS35 pathway (22).

A current limitation in the dissection of endolysosomal function is the lack of approaches for analysis of specific organelles within the endolysosomal system in neuronal systems. Here, we report the development of a toolkit for profiling a population of early/sorting endosomes, as well as lysosomes (23), in human ES cell (hESC)-derived cortical-like iNeurons. We target a subpopulation of early endosomes marked by endogenously tagged EEA1 (Early Endosome Antigen 1) protein, which represents one of the earliest populations formed after endocytic vesicle uncoating. EEA1 is a peripheral membrane protein containing a C-terminal FYVE domain that binds phosphatidylinositol 3-phosphate, and an N-terminal zinc finger domain that interacts with RAB5. These interactions underly an early endosomal tethering function that facilitates vesicle fusion (24). In neurons, EEA1 is found in the cell body but can also be found on somatodendritic endosomes as well as on endosomes at the nerve terminal (25, 26). Given the proximity of this population to uncoating, this “Endo-IP” approach (27) allows detection of the largest repertoire of endocytic cargo, prior to degradation during maturation to lysosomes. Our work revealed broad remodeling of endocytic cargo during in vitro neurogenesis, reflecting programmed expression of a diverse repertoire of PM proteins involved in numerous neuronal functions. In contrast, lysosomes display a much simpler proteome rich in hydrolytic enzymes, reflecting both cargo sorting for retrograde transport or degradation during endolysosomal maturation. We demonstrate that this approach can be used to profile early endosomes dynamics, such as during iNeuron depolarization. Finally, we present a functional landscape of endocytic cargo, as well as a compendium of candidate cargo sorting motifs and structural predictions for two major sorting motifs linked with SNX27-Retromer and SNX17-Retriever (21, 28–31). This work provides the tools for mechanistic interrogation of pathways controlling cargo selection and the impact of neurodegenerative disease risk alleles on this process.

## Results and Discussion

### Genetic Tagging of EEA1 and TMEM192 in Human Stem Cells.

**Fig. 1.**
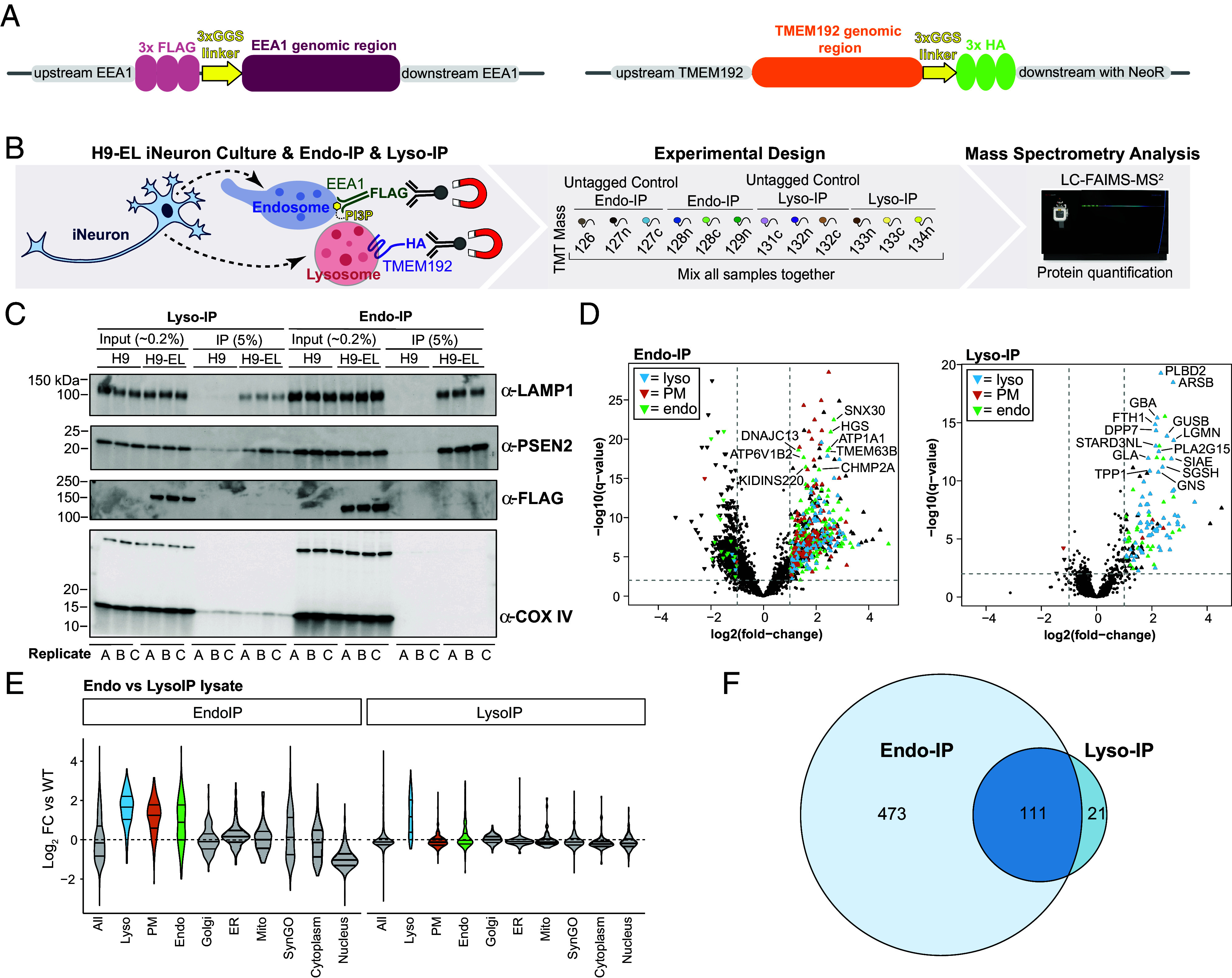
Toolkit and validation of Endo-IP and Lyso-IP for organelle proteomics in hESC-derived iNeurons. (*A*) Diagram of endogenous tagging of the N-terminus of EEA1 and the C-terminus of TMEM192 with 3XFLAG and 3XHA tags using CRISPR-Cas9. (*B*) Schematic of TMT-based proteomic analysis of Endo-IP and Lyso-IP from H9-EL-derived day 21 iNeurons. EEA1-positive endosomes are captured with α-FLAG conjugated to magnetic beads and TMEM192-positive lysosomes are similarly captured with α-HA antibodies. Purified EEA1-positive or TMEM192- positive membranes were eluted from the beads with a mild detergent, and proteins were digested with LysC and trypsin. The resulting peptides were labeled with 12-plex TMT and analyzed by mass spectrometry (MS). (*C*) Immunoblotting of the indicated extracts or immunoprecipitation (IP) samples using α-LAMP1 and α-PSEN2 to indicate endolysosomes and α-FLAG to detect FLAG-EEA1. α-COXIV was used as a control for nonspecific capture of mitochondria. “*A*, *B*, and *C*” refer to independent replicate experiments. The percentage of input and IP loaded on the gel are indicated. FLAG-EEA1 is retained on the affinity resin and is therefore not detected in the immunoblot of the Endo-IP elution. (*D*) Volcano plots of log_2_ fold change (FC) for tagged versus untagged (control) iNeurons versus -log10(q-values) are plotted. Proteins annotated as lysosomal (cyan), PM (orange), or endosomal (green) are indicated. (*E*) Violin plots of log_2_ FC relative to WT (untagged control) iNeurons for various groups of proteins. (*F*) Venn diagram for overlap of proteins enriched in the Endo-IP and Lyso-IP from iNeurons.

To purify endolysosomal compartments from human neurons, we adopted the Endo-IP approach using EEA1 protein as an affinity handle, incorporating three copies of the FLAG epitope (27) (Fig. 1*A*). Unlike gradient fractionation of endosomes which captures many classes of endosomes (32), the EEA1 affinity handle allows capture of a small population of endosomes that is expected to be enriched in newly endocytosed cargo. To avoid any effects of FLAG-EEA1 overexpression, we endogenously tagged both EEA1 copies using CRISPR-Cas9 in hESCs harboring an inducible AAVS1-NGN2 neurogenesis driver (33–35) (*SI Appendix*, Fig. S1*A*). In parallel, we similarly edited FLAG-EEA1 in hESCs previously engineered to contain the Lyso-IP affinity handle—a triple HA epitope fused with the C-terminus of TMEM192 (in heterozygous form, see *Materials and Methods*) (Fig. 1*A* and *SI Appendix*, Fig. S1*B*). These cell lines were validated by sequencing and immunoblotting of cell extracts from hESCs and day 21 iNeurons (*SI Appendix*, Fig. S1 *A*–*D*). We refer to singly and doubly tagged cells as “H9-E” and “H9-EL”, with “E” referring to Endo-IP and “EL” referring to Endo- and Lyso-IP.

To ensure proper localization of Endo- and Lyso-IP tags, we performed immunofluorescence in combination with confocal microscopy on day 21 to 23 iNeurons. As expected, α-FLAG and α-EEA1 signals displayed a very high degree of colocalization (Mander’s coefficient >0.75) (*SI Appendix*, Fig. S1*F*). Consistent with EEA1 colocalization with a subset of early/sorting endosomes, small mean Mander’s coefficients were observed for α-RAB5 and α-FLAG (0.1 to 0.28) or α-RAB5 and α-EEA1 (0.2 to 0.24) in both H9-E and H9-EL cell lines (*SI Appendix*, Fig. S1 *E* and *F*). The extent of colocalization between EEA1 and RAB5 was slightly reduced in H9-E and H9-EL tagged cells compared to WT untagged controls (mean Mander’s coefficients of 0.2 to 0.24 vs 0.36 to 0.4) (*SI Appendix*, Fig. S1 *E* and *F*). For Lyso-IP tagged iNeurons, TMEM192-HA puncta partially colocalized with the lysosomal marker LAMP1 with Mander’s coefficients in the range of 0.3 to 0.7. Mander’s coefficients were not significantly different between Lyso-IP-tagged and untagged iNeurons (mean Mander’s coefficients of 0.4) (*SI Appendix*, Fig. S1 *E* and *F*). The extent of RAB5 and EEA1 colocalization was in agreement with the broad range reported previously in HEK293 (27) and HeLa (36) cells. Cell line variability in the distribution of EEA1 between early/sorting endosomes and late endosomes has been observed previously (36). Taken together, these data indicate the validity of these cells for analysis of EEA1-positive populations of endosomes and of TMEM192-positive lysosomes.

### Benchmarking Dual-Tagged EL Cells for Organelle Proteomics.

To initially benchmark H9-EL cells for organelle IP, we converted control (untagged H9) and H9-EL cells to day 21 iNeurons in biological triplicate and performed Endo- and Lyso-IPs followed by either immunoblotting or 12-plex Tandem Mass Tagging (TMT)-based MS (Fig. 1*B*). Immunoblotting of Endo- or Lyso-IP samples demonstrated selective enrichment of LAMP1 and PSEN2, with no enrichment of COX IV as a mitochondrial marker (Fig. 1*C*). We note that the FLAG-tagged EEA1 protein is retained on the affinity resin under the gentle elution conditions used here and is therefore not detected in the elution from the immunoprecipitate (Fig. 1*C*). Proteomic analysis revealed that Lyso-IP samples were substantially enriched in known lysosomal proteins, including catabolic enzymes (GNS, GUSB, GBA, GLA), among many others (Fig. 1 *D* and *E* and *SI Appendix*, Fig. S2*A*, and Dataset S1). Indeed, of the 132 proteins with Log_2_ FC>1.0, 104 were known lysosomal proteins and 7 others were annotated as secreted or PM-localized (Fig. 1*D* and *SI Appendix*, Fig. S2*D*). Moreover, analysis of several subcellular compartments demonstrated a high degree of selectivity in the enrichment of lysosomal proteins (Fig. 1*E* and *SI Appendix*, Fig. S2*B*). Of the 91 proteins enriched in Lyso-IPs from HEK293 cells, 56 were enriched in iNeurons (*SI Appendix*, Fig. S2*C*). Interestingly, the overall number of proteins enriched in the Endo-IP of iNeurons was ~4 times larger than with Lyso-IP and was substantially populated with PM proteins in addition to numerous proteins annotated as endosomal or lysosomal (Fig. 1 *D*–*F* and *SI Appendix*, Fig. S2 *A* and *B*, and Dataset S1). As expected, proteins found in common in both Endo-IP and Lyso-IP are typically core components of the endolysosomal system, including BORCS6, LAMTOR2, and ATP13A2 (Fig. 1*F* and *SI Appendix*, Fig. S2*D*). These results indicate the feasibility of endosome and lysosome proteome profiling in iNeurons and the capacity of Endo-IP to capture protein cargo.

### Proteomic Landscape of EEA1-Positive Endosomes During In Vitro Neurogenesis.

**Fig. 2.**
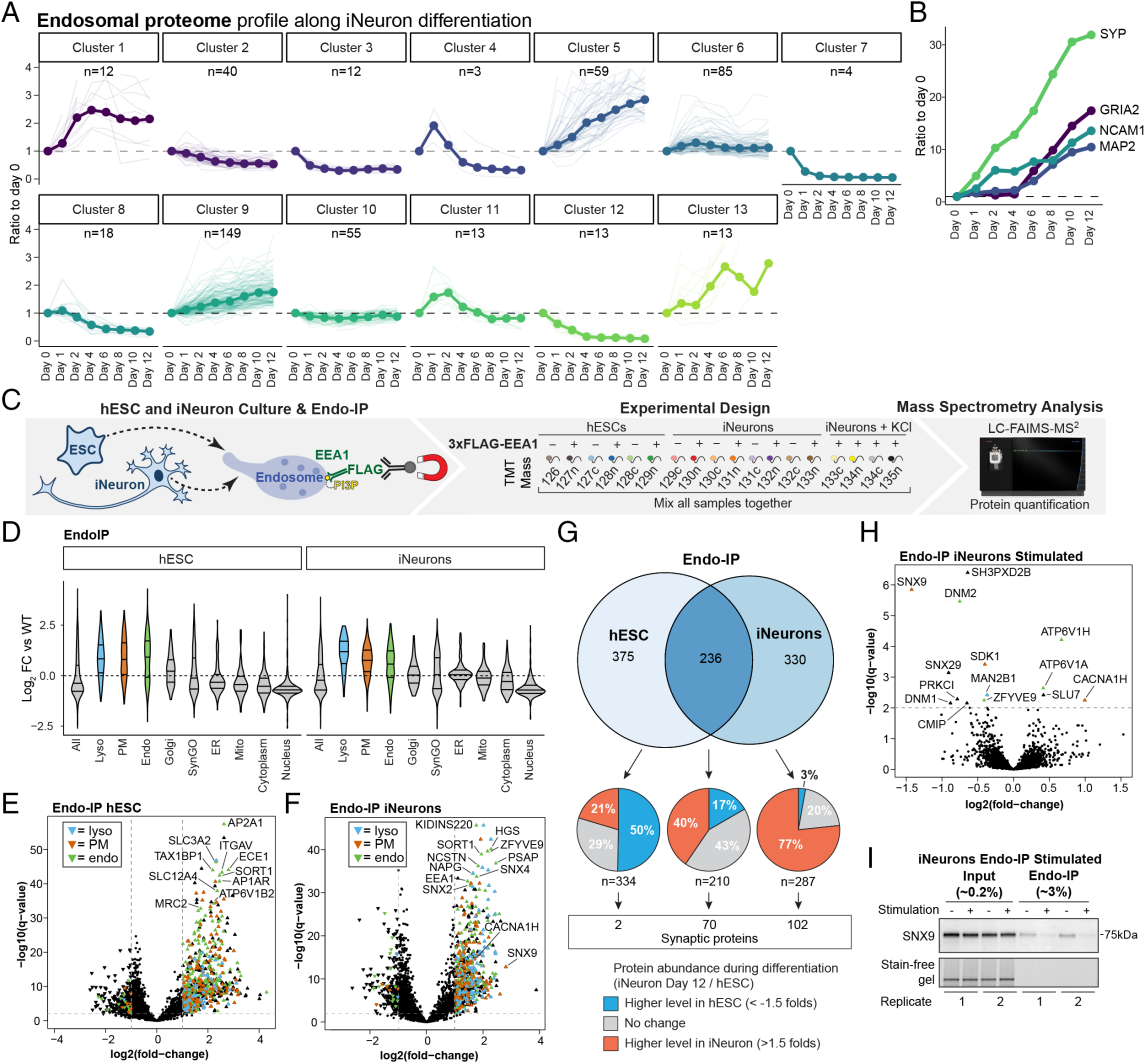
Proteomic Landscape of EEA1-positive Endosomes in hESCs and iNeurons. (*A*) Alterations in endosomal proteins during in vitro differentiation of hESCs to iNeurons, displayed as clusters identified based on patterns of expression from our previously reported TMT-based proteomic analysis of H9 cells using the NGN2 driver (34). (*B*) Analogous to panel A for 4 markers of neurogenesis (SYP, GRIA2, NCAM1, and MAP2). (*C*) Schematic of our approach for quantitative analysis of the hESC and day 21 iNeuron endosomal proteome using the Endo-IP method for H9-E cells, and parental H9 cells as a control in triplicate or quadruplicate, as well as quadruplicate iNeurons stimulated with KCl (50 mM, 10 min). Purified EEA1-positive membranes were eluted from the beads with a mild detergent, and proteins were digested with LysC and trypsin. The resulting peptides were labeled with 14-plex TMT and analyzed by MS. (*D*) Violin plots of log_2_ FC relative to WT (untagged control) cells for various groups of proteins found in Endo-IPs from hESCs or day 21 iNeurons. (*E* and *F*) Volcano plots of log_2_ FC for tagged versus untagged (control) hESCs (panel *E*) or iNeurons (panel *F*) versus −log10(q-values) are plotted. Proteins annotated as lysosomal (cyan), PM (orange), or endosomal (green) are indicated. The dashed lines indicate the log_2_ FC cutoff of 1.0 and -log10(q-value) cutoff of 2 (q-value of 0.01). (*G*) Comparison of proteins identified by Endo-IP in hESCs and day 21 iNeurons (upper Venn diagram). Pie charts display the proportion of identified proteins with abundance changes in either the hESC or iNeuron state, based on (34). The number of proteins present in SynGO is indicated. (*H*) Volcano plot of Log_2_ FC for Endo-IPs from depolarized iNeurons (50 mM KCl, 10 min) relative to untreated iNeurons. Proteins displaying significant alterations in abundance are indicated. (*I*) Immunoblot validation of reduced SNX9 levels in Endo-IPs in parallel with the scheme in panel *C*. Stain-free gels indicate equal input levels for each sample.

Our previous studies have demonstrated large-scale proteome remodeling during NGN2-driven conversion of hESCs to iNeurons in vitro, including the induction of numerous factors that drive the neurogenesis program (34). A total of 476 endolysosome-related proteins (see *SI Appendix*, *Materials and Methods* and Dataset S2) were categorized in clusters based on their abundance profile during a 12-d differentiation, revealing that most proteins dramatically increased in abundance during neurodifferentiation (Fig. 2*A*). Moreover, many proteins annotated in Synaptic Gene Ontology (SynGO)—an evidence-based and curated database for synaptic proteins, a subset of which are also know to localize on endosomes (37)—also displayed similar alterations in abundance during differentiation (*SI Appendix*, Fig. S3*A* and Dataset S2). This includes markers for synaptic vesicles (SYP), postsynaptic receptor (GRIA2), dendrites (MAP2), and adhesion (NCAM1) (Fig. 2*B*).

To identify endocytic factors and neuron-specific cargo, we performed TMT-MS on triplicate or quadruplicate Endo-IPs from H9 control or H9-E cells in either the stem cell state or iNeurons at day 21 of differentiation (Fig. 2*C*). As expected, immunoblotting of iNeuron Endo-IP samples revealed enrichment for LAMP1 as an endolysosomal marker, but not Golgi (GOLGA1) or ER (CALR) markers (*SI Appendix*, Fig. S3*B*). The reproducibility of the method was assessed by comparing two independent Endo-IP datasets, which revealed extensive overlap of enriched proteins identified and reproducible protein levels (*SI Appendix*, Fig. S3 *C* and *D*). Consistent with the results described above, Endo-IPs from both hESC and iNeuron populations displayed enrichment of numerous endolysosomal and PM proteins, but no enrichment in other cellular compartments (Log_2_ FC ≥ 1.0, q-value ≤ 0.01) (Fig. 2 *D*–*F* and *SI Appendix*, Fig. S3*E*). From the 611 and 566 proteins enriched in Endo-IPs in hESCs and iNeurons, respectively, 236 were common to both cell types (Fig. 2*G*). These included nutrient transporters (ECE1, SLC3A2), subunits of sorting complexes (AP2A1, VPS35, SORT1, SNX4, SNX2, and NAPG), v-ATPase subunits (ATP6V1B1 and ATP6V1E1), signal transduction factors (ZFVYE9), autophagy pathways components (TAX1BP1, GABARAP, GABARAPL1, and GABARAPL2), and other established endosomal factors or cargoes (PSAP, EEA1, HGS, and NCSTN) (Fig. 2*G* and Dataset S2). Enriched proteins that were selective for one or the other cell state tended to correlate with the relative levels of expression; 79% of hESC-selective proteins had higher abundance (50%) or were of equal abundance (29%) in hESCs while 97% of iNeuron-selective proteins had higher abundance (77%) or were of equal abundance (20%) in iNeurons (Fig. 2*G*). Similarly, 102 synaptic proteins were identified exclusively in iNeurons, while only 2 and 70 were identified only in hESCs or both hESCs and iNeurons, respectively. In addition, the fraction of transmembrane segment-containing proteins found specifically in hESCs or in iNeurons was comparable (54 and 58%, respectively). Taken together, these data indicate that hESCs and iNeurons have partially nonoverlapping EEA1-positive endosomal proteomes.

### Endo-IP During Membrane Depolarization in iNeurons.

The endosomal system is highly dynamic and rapidly modulates the cell surface proteome. To capture the reorganization of proteins associated with EEA1-positive endosomes during neuronal dynamics, we tested whether alterations in protein levels could be detected in response to neuronal depolarization (Fig. 2*C*). Endo-IPs from iNeurons depolarized for 10 min with KCl revealed a small number of proteins whose abundance either increased or decreased compared to unstimulated iNeurons (Fig. 2*H* and Dataset S2). Among the proteins that increased in abundance was CACNA1H (also called Cav3.2), a voltage-sensitive calcium channel that gives rise to T-type calcium currents and is linked with epilepsy (38). In contrast, the levels of proteins involved in endocytosis (SNX9, DNM1, and DNM2) were reduced. Sorting nexin SNX9 was the most dramatically downregulated protein following depolarization (Fig. 2*H* and Dataset S2), which was validated by immunoblotting (Fig. 2*I*). Interestingly, SNX9 is highly enriched in Endo-IPs from iNeurons but not in hESCs (Fig. 2 *E* and *F* and Dataset S2). Taken together, these data indicate that Endo-IPs can be used to assess potentially rapid signal-dependent neuronal alterations in EEA1-positive endosomal proteins. This result parallels our previous experiments demonstrating the capture of endocytic cargo within 5 min of ligand stimulation in HEK293 cells (27).

### Repertoire of Receptor and Synaptic Protein Cargo in iNeurons.

**Fig. 3.**
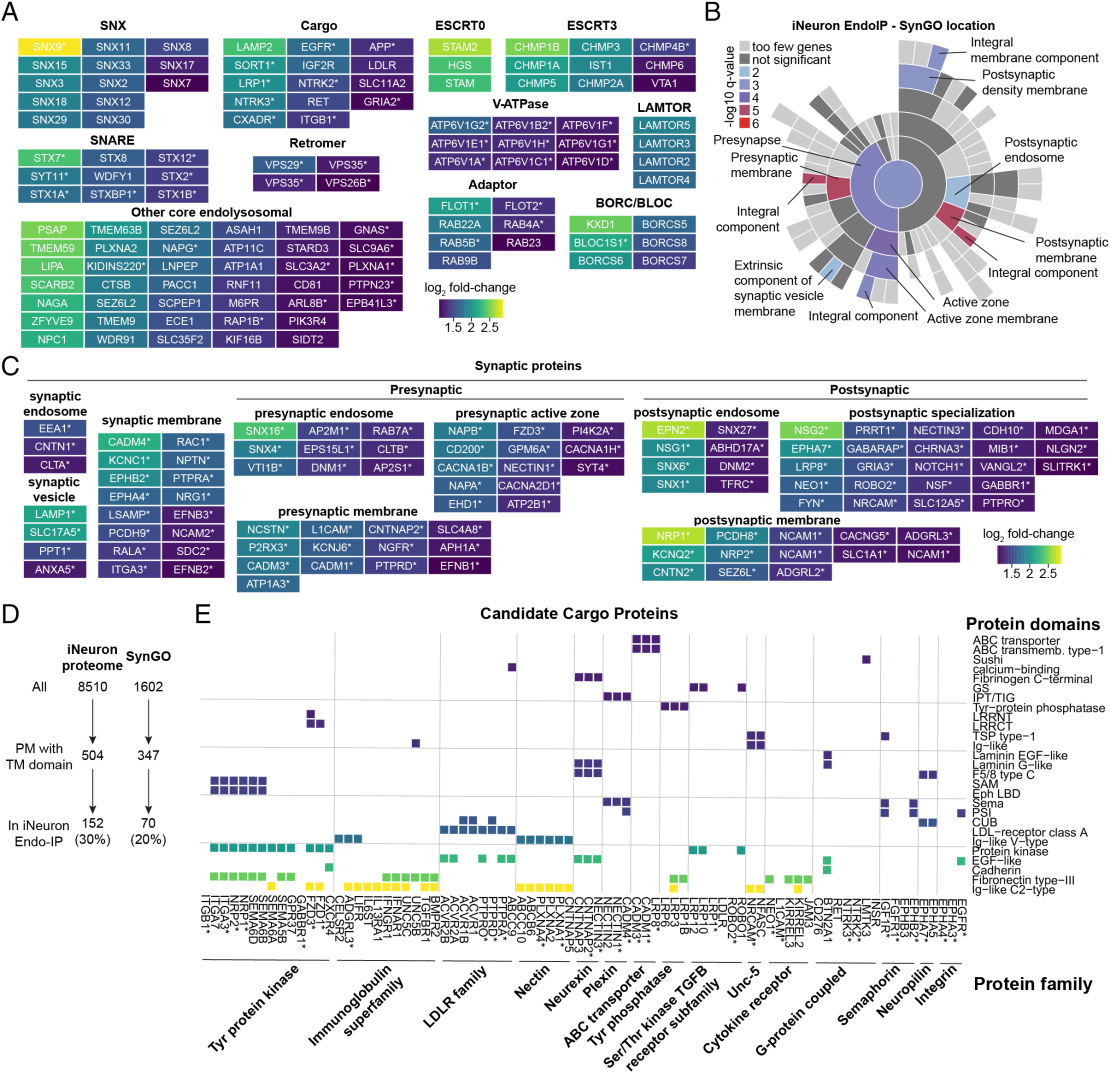
Repertoire of Receptor and Synaptic Protein Cargo in iNeurons. (*A*) Functional categories of endosomal proteins identified by Endo-IP in day 21 iNeurons. Color code refers to the extent of enrichment in Endo-IP (log_2_ fold-change), as indicated by the scale bar. Proteins present in SynGO are indicated with an asterisk. (*B*) Enrichment of iNeuron Endo-IP proteins based on SynGO location. Color code corresponds to the log_10_ of q-value for individual categories of SynGO proteins. (*C*) Categories of synaptic proteins identified as being enriched in Endo-IPs from day 21 iNeurons. (*D*) Relative enrichment of iNeuron proteome (34) and SynGO proteome within the Endo-IP from day 21 iNeurons. (*E*) Classification of a subset of transmembrane proteins and their associated extracellular domains and protein families identified as enriched in Endo-IPs from day 21 iNeurons. Colored squares reflect the presence of the indicated domain within individual proteins. Proteins are organized based on their presence within the indicated protein family. An asterisk indicates that the protein is present in SynGO.

To characterize the endolysosomal proteins identified in iNeurons, we first organized Endo-IP enriched proteins into broad functional categories, encompassing many core endolysosomal functions, including sorting (Retromer, ESCRT), membrane fusion (SNAREs), positioning (BORC), and numerous core endolysosomal proteins (Fig. 3*A*). Many of these proteins, indicated by asterisks, are also present in SynGO, consistent with known roles for endosomal vesicle fusion and vesicle positioning within the synaptic compartment (Fig. 3*B*). SynGO analysis also revealed a significant enrichment of additional synaptic proteins in the iNeuron Endo-IP not previously linked with core endosomal functions, including several categories of pre- and postsynaptic subcellular locations, and functional categories (e.g., synaptic organization, signaling, and transport) (Fig. 3 *B* and *C* and *SI Appendix*, Fig. S4 *A* and *B*). Further mining of the synaptic proteins revealed numerous proteins linked with synaptic endosomes and synaptic membranes that most likely represent endocytic cargo, featuring diverse transmembrane receptors and adhesion proteins (Fig. 3*C*).

To further understand the repertoire of candidate cargo in iNeurons, we examined all proteins harboring one or more transmembrane segments which are also annotated as localized in the cell membrane based on Uniprot. We found 504 and 347 of such proteins in iNeurons and SynGO, of which 152 and 70 were also identified in Endo-IP, respectively (Fig. 3*D*). This indicates that 30% of all PM proteins in iNeurons and 20% of PM proteins with annotated synaptic localization are candidate cargo for endocytosis. We next identified 241 candidate cargo specifically in our Endo-IP in iNeurons, applying the same transmembrane and cell membrane localization criteria (Dataset S2), and further categorized candidate cargo by their annotated domains and protein families (Fig. 3*E* and *SI Appendix*, Fig. S4*C*). Of the 80 candidate cargo containing both an annotated domain and protein family (including 34 in SynGO), more than a dozen types of extracellular domains were represented. These included Ig-like C2-type, fibronectin, EGF-like, and protein kinase domains, found in members of the Nectin, low-density lipid receptor (LDLR), Neurexin, immunoglobulin, and tyrosine protein kinase families of proteins (Fig. 3*E*). Additionally, 89 further candidate cargo (including 24 in SynGO) with annotations for either a domain or a protein family, including channels, transporters, peptidases, and tetraspanin proteins, were identified (*SI Appendix*, Fig. S4*C*). Given the kinetics of protein recycling or degradation upon endocytosis (on the order of minutes), the diversity of proteins identified with Endo-IP enrichment suggests extensive ongoing remodeling of the PM proteome during in vitro neurogenesis.

### Matching Candidate Cargo with Sorting Motifs.

**Fig. 4.**
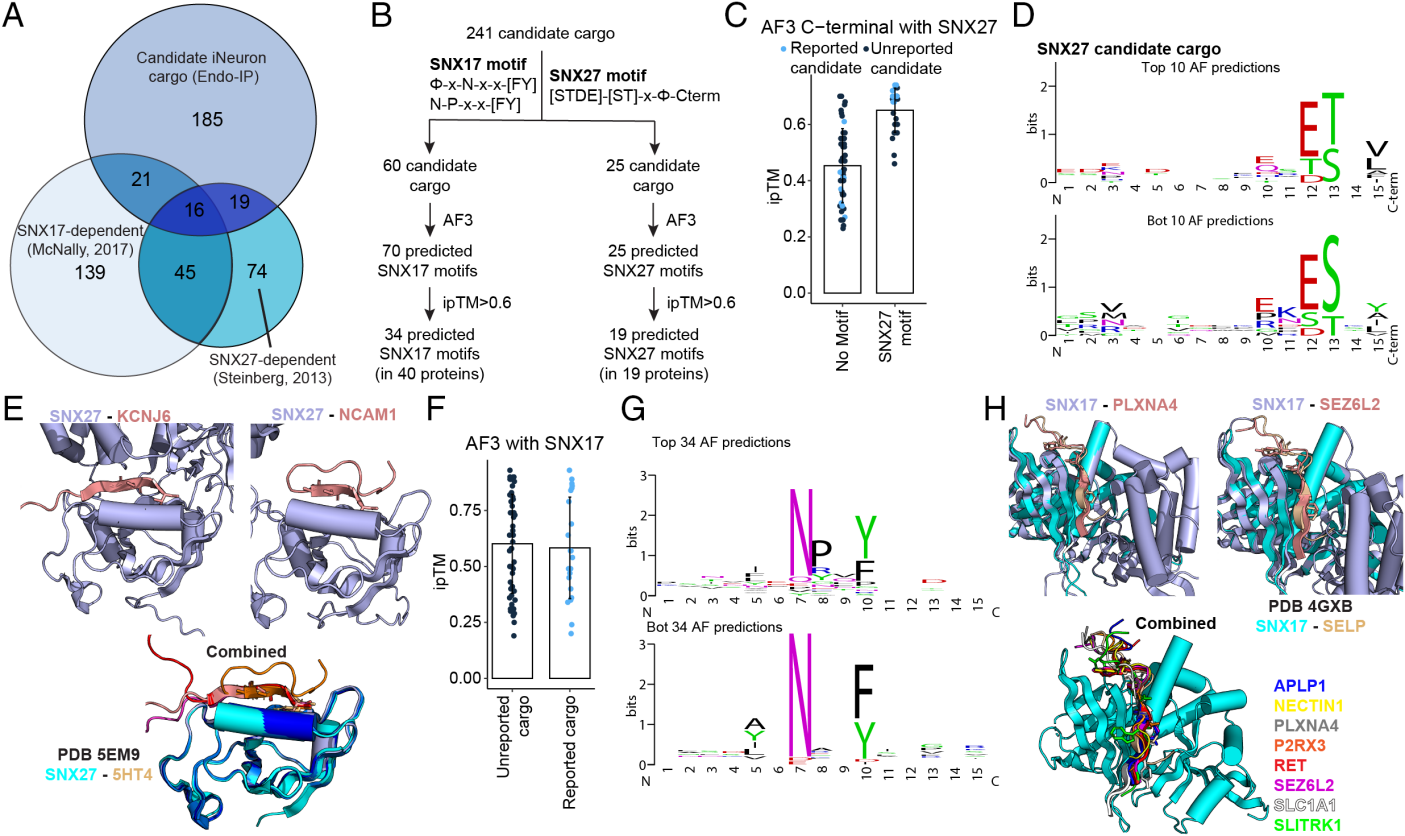
Matching candidate cargo with SNX17 and SNX27 sorting motifs. (*A*) Comparison of the proteins identified by Endo-IP in day 21 iNeurons with proteins whose cell surface levels depend on SNX17 or SNX27 and proteins that associate with SNX27 (22, 29). The number of proteins in each category is indicated. (*B*) Scheme summarizing the search for SNX17 or SNX27 sorting motifs in 241 candidate cargo. Candidate motifs were subjected to predictions using AF3 and the number of proteins with ipTM scores >0.6 indicated. (*C*) Plot of ipTM values for AF3 predictions involving proteins with or without candidate SNX27 motifs. Light blue dots represent proteins reported as candidates for SNX27 cargo. (*D*) Motif plots for *Top* 10 and *Bottom* (Bot) 10 AF3 predictions. (*E*) Structural predictions for candidate iNeuron cargo and the PDZ domain of SNX27. C-terminal motifs are shown in salmon and SNX27 in light blue. The structure of SNX27-5HT4 (PDB: 5EM9) is overlaid in cyan and brown. (*F*) Plot of ipTM values for AF3 predictions involving candidate proteins with SNX17 motifs. Light blue dots represent proteins reported as candidate SNX17 cargo. (*G*) Motif plots for *Top* 34 and *Bottom* (Bot) 34 AF3 predictions. (*H*) Structural predictions for candidate iNeuron cargo and the FERM domain of SNX17. SNX17-motif peptide predictions are in light blue (SNX17) and salmon (PLXNA4 and SEZ6L2 motifs) or other colors as indicated for additional motifs. The structure of SNX17-SELP (30) (PDB: 4GXB) is overlaid in cyan and brown.

Early endosomes function as sites of cargo sorting for recycling to the PM or Golgi and also further mature to late endosomes, whereas cargo destined for turnover are internalized into intralumenal vesicles for subsequent degradation in the lysosome (4). Retromer and Retriever function as sorting complexes in conjunction with a variety of sorting nexins, including SNX27 and SNX17, respectively (22, 29, 30, 39). Mutations in the VPS35 subunit of Retromer have been linked with Parkinson’s disease, while mutations in the CCDC22 subunit of Commander (Retriever plus the CCC complex) are found in Ritscher-Schinzel syndrome (21, 40). To initially examine the relationship between candidate cargo identified in the iNeuron system and known SNX17 and/or SNX27 cargo, we used previously reported datasets (22, 29) to ask to what extent our candidate cargo were among proteins whose abundance on the PM was altered in HeLa cells depleted of SNX17 or SNX27. For this purpose, we employed the 241 proteins in our iNeuron dataset that contained at least one transmembrane domain and that were annotated in Uniprot as being localized in the cell membrane (Dataset S3). The majority (75%) of iNeuron proteins were not previously identified in the SNX17/SNX27 studies, potentially reflecting cell type diversity, while 16 to 21 proteins (6.5 to 8.5%) were common to two or three datasets (Fig. 4*A*). The finding that many candidate cargo in iNeurons were absent from known cargo led us to examine these proteins for the presence of sorting motifs.

SNX27 uses its PDZ domain to bind [S/T]-x-Φ-C-terminal motifs in cargo (in which Φ represents any hydrophobic residue as the C-terminal residue in the candidate protein) (22, 28, 41, 42). This core interaction is frequently preceded by one or more acidic residues or phosphorylated Ser/Thr residues in a manner that enhances binding (31). In contrast, SNX17 uses its FERM (4.1/ezrin/radixin/moesin) domain to bind to ΦxNxx[F/Y] and related motifs (where Φ is a hydrophobic amino acid) in cargo (30). Motif searches revealed 25 and 60 candidate cargo containing potential SNX27 and/or SNX17 interaction motifs, respectively (Fig. 4*B*). For the 25 SNX27 candidates, we screened their C-termini as 15 amino acid peptides for interaction with the PDZ domain of SNX27 using AF3, within the context of full-length SNX27 (43). In parallel, we screened a set of 47 potential SNX17 cargo that lacked an apparent SNX27 binding motif as controls for AF3 predictions (Fig. 4*B*). We identified 19 candidate cargo with AF3 ipTM scores >0.6 (Fig. 4*C* and Dataset S3). In contrast, the majority of control sequences lacking an SNX27 motif had ipTM scores <0.6. Importantly, of the candidate SNX27 cargo that were also identified previously in HeLa cells, the majority had ipTM scores of >0.6 (Fig. 4*C*). As expected, the motifs identified among the top 10 and bottom 10 AF3 predictions conform to the canonical SNX27 sorting motif (Fig. 4*D*). Examples of SNX27 PDZ domain-candidate sorting motif predictions for KCNJ6, KCNJ12, NCAM1, and NCAM2 are shown independently and together with the previously determined structure of SNX27 and 5HT4 (PDB 5EM9) (31) (Fig. 4*E* and *SI Appendix*, Fig. S5*A*).

Similarly for candidate SNX17 cargo, a total of 70 potential cargo sorting motifs in 60 candidate proteins were screened by AF3, leading to the identification of 34 candidate SNX17 sorting motifs in 40 proteins with ipTM scores >0.6 (Fig. 4 *B* and *F* and Dataset S3). The distribution of ipTM scores for known and previously unidentified candidate cargo were similar, with many known cargo having ipTM scores less than 0.6 (Fig. 4*F*). As expected, the motif identified for the top scoring cargo fit the consensus for SNX17 FERM binding proteins (Fig. 4*G*). The predicted structures for several candidate cargo closely matched the previously determined structure for SNX17 and SELP (PDB: 4GXB) (30) (Fig. 4*H* and *SI Appendix*, Fig. S5*B*). Taken together, these data uncovered unique neuronal-specific cargo in human neurons and showcase how Endo-IP can be used to enrich and identify candidate cargo for specific sorting pathways.

## Conclusions

Stem cell–derived neurons have emerged as important tools for understanding the basic cell biology and functions of genes linked with neurogenesis. Multiple lineage drivers have been developed, affording the potential to create distinct classes of iNeurons with unique properties (35, 44–46). iNeurons maintain many features of animal-derived neurons, including intact axonal and dendritic trafficking pathways, and reliance on organelle quality control systems such as autophagy to remove damaged or superfluous organelles (16, 47, 48). In addition, efforts to generate diverse mutations linked with neurodegenerative diseases have been initiated, with the long-term goal of understanding how risk alleles linked with specific diseases alter critical trafficking and quality control pathways (49). With these advances in mind, we set out to develop tools to isolate early endosomes and lysosomes using genetically tractable hESC-derived cortical-type iNeurons. We demonstrate the utility of endogenously tagged EEA1 and TMEM192 genes with FLAG and HA epitopes, respectively, for isolation of early endosomal and lysosomal populations from day 21 cortical-type iNeurons, and for profiling early endosomal populations in stimulated iNeurons. Thus, overexpression of tagged affinity handles, as employed previously (23, 27), is not required for effective capture of organelles.

Our studies reveal a diverse array of candidate endocytic cargo identified by Endo-IP, including many classes of single and multipass transmembrane proteins functioning in a variety of pathways associated with neurogenesis. Through systematic motif analysis coupled with AF3-based predictions, we provide a compendium of candidate cargo for SNX27- and SNX17-based sorting machinery, which function with Retromer and Retriever complexes, respectively (4, 21). Interestingly, our approach identified several candidate cargo for SNX17 or SNX27, including several proteins linked with neurogenesis and neuronal function (e.g., PLXNA4, P2RX3, RET, SEZ6L2, SLC1A1, SLITRK1) (Fig. 4*H* and *SI Appendix*, Fig. S5 *A* and *B*). Further directed studies in the context of sorting mutants are required to validate specific candidate motifs and associated machinery, and any spatial aspects of endocytic selectivity (12), but our analysis provides a broad landscape for such studies. Moreover, we envision that distinct sets of cargo will be identified in distinct types of iNeurons, in the context of activation, or in the presence of glial cells which promote the formation of functional synaptic compartments. Additionally, our compendium of candidate cargo will facilitate studies designed to understand spatial control of endocytosis. Taken together, our toolkit enables profiling diverse aspects of the endolysosomal components to further understand the system in physiological and neurodegenerative conditions.

## Materials and Methods

Detailed procedures related to cell culture, gene editing, biochemical procedures, MS, microscopy, and flow cytometry are described in *SI Appendix*, *Materials and Methods*. Statistical analyses were performed using MSstats and ggplot. All error bars represent SEM, and statistical significance was determined by *t* tests, as specified in the corresponding figure legends.

## Supplementary Material

Appendix 01 (PDF)

Dataset S01 (XLSX)

Dataset S02 (XLSX)

Dataset S03 (XLSX)

## Data Availability

Cell lines reported here will be made available upon request with no restrictions. Proteomic data (.RAW files) are available via ProteomeXchange with identifier PXD055978 (50). The key resource table, uncropped blots, light microscopy images, data analysis scripts, source data for colocalization bar graphs, AF3 prediction PDB files, and supplementary datasets associated with this publication are available from Zenodo.org: https://doi.org/10.5281/zenodo.13955416, https://doi.org/10.5281/zenodo.13882568, https://doi.org/10.5281/zenodo.13882797, https://doi.org/10.5281/zenodo. 13885953, https://doi.org/10.5281/zenodo.13885853, https://doi.org/10.5281/zenodo.13882583, https://doi.org/10.5281/zenodo.13984203 (51–57). All other data are included in the manuscript and/or supporting information.
